# Editorial: Enriching genomic breeding with environmental covariates, crop models, and high-throughput phenotyping

**DOI:** 10.3389/fgene.2024.1391938

**Published:** 2024-03-13

**Authors:** Alencar Xavier, Katy M. Rainey, Kelly R. Robbins

**Affiliations:** ^1^ Corteva Agrisciences, Johnston, IA, United States; ^2^ Department of Agronomy, Purdue University, West Lafayette, IN, United States; ^3^ College of Agriculture and Life Sciences, Cornell University, Ithaca, NY, United States

**Keywords:** enviromics, genomic prediction, phenomics, crop models, plant breeding

Plant breeding stands at the intersection of innovation, technology, and the ever-growing demand for sustainable agricultural practices. In this special edition of Frontiers in Genetics, we delve into the intricate landscape of genomic breeding enriched by environmental covariates, crop models, and high-throughput phenotyping. Our goal is to showcase the integration of diverse data sources and methodologies, providing a comprehensive view of the advancements in this field.

The six articles Tolley et al.; López et al.; Jackson et al.; Boatwright et al.; Cooper et al.; Persa et al. (2023) comprising this issue address key aspects of modern plant breeding, bringing together insights from genomics, enviromics, and phenomics.

Our goal in this special edition is to offer a platform for researchers to share their experiences, methodologies, and insights. We sought contributions that not only identify knowledge gaps but also evaluate methodological approaches suitable for analyzing complex data and integrating various sources of information. The proof-of-concept breeding applications presented in these articles showcase the successful intersection of high-throughput or longitudinal phenotyping, genomic-assisted breeding, environmental information, and crop growth modeling.

This special issue of Frontiers in Genetics is a testament to the dynamic and interdisciplinary nature of modern plant breeding. It serves as a valuable resource for researchers, practitioners, and policymakers, providing a glimpse into the exciting future of agriculture where data-driven approaches play a pivotal role in shaping resilient and high-yielding crop varieties. We extend our sincere appreciation to the authors, reviewers, and the editorial team for their contributions to this enriching exploration of the frontiers in genomic breeding. May this collection inspire further collaboration and innovation in the quest for sustainable and productive agriculture.

In recent years, the field of plant breeding has undergone a remarkable transformation, catalyzed by the integration of cutting-edge technologies and data-driven approaches. This special edition of Frontiers in Genetics showcases a compilation of six articles at the forefront of this revolution, focusing on the convergence of genomics, environmental covariates, crop models, and high-throughput phenotyping. The collective aim is to unravel the intricate interplay between genetic factors and environmental influences, ultimately advancing the precision and efficiency of modern breeding strategies.

Tolley et al. kick-start the discourse by delving into the genomic prediction and association mapping of maize grain yield across diverse environments. Employing reaction norm models, their study offers insights into the complex genotype-by-environment interactions, providing a robust foundation for informed breeding decisions in variable agricultural landscapes.

López et al. contributes a thought-provoking exploration on the role of feature selection methods in enhancing genomic prediction accuracy by selecting relevant environmental covariables. This article navigates the nuanced terrain of data processing, shedding light on methodologies that refine predictions and augment the efficacy of breeding programs.

Jackson et al. embark on a journey into winter wheat, seamlessly integrating phenomic and genomic data for yield prediction across multiple locations. By harmonizing high-throughput phenotyping with genomic insights, this work pioneers a holistic approach to breeding, fostering a deeper understanding of the genetic architecture underlying adaptive responses to diverse environmental conditions.

Shifting the focus to sorghum, Boatwright et al. unravel the functional genomic effects of indels through Bayesian genome-phenome wide association studies. Their contribution marks a pivotal step in deciphering the role of genetic variations in shaping phenotypic outcomes, offering valuable cues for targeted breeding interventions.

Cooper et al. extend the traditional breeder’s equation to take aim at the target population of environments. By incorporating insights from quantitative genetics, this article proposes a novel framework to navigate the complex genotype-environment landscape, providing breeders with a strategic guide to optimize selection decisions.

Wrapping up this special edition, Persa et al. (2023) address the challenges posed by sparse testing designs in soybean populations. Their work focuses on improving predictive ability, offering practical solutions to enhance the robustness of breeding programs in the face of limited resources and data constraints.

Collectively, these articles represent a tapestry of advancements at the intersection of genomics, environmental covariates, crop models, and high-throughput phenotyping. As we navigate this era of unprecedented technological integration, the methodologies and proof of concepts presented in this special edition serve as beacons, guiding researchers and practitioners toward a future where data-driven precision in plant breeding becomes the norm. The contributors not only bridge existing knowledge gaps but also inspire a broader dialogue on the deployment of novel technologies in the quest for sustainable and resilient crops that can thrive in diverse and challenging environments.
